# 

**Published:** 1894-12-22

**Authors:** 


### CONTENTS.

**The Flowing Tide of Christmas Charity** - - - - 199
**Gastrostomy** - - - - - - - - - - - 200
**Modern Medico-Psychology and Psychiatry.** By T. S. Clouston, M.D. XIII.—States of Defective Control and of Morbid and Uncontrollable Impulse - - - - - - 201
**Medical Progress and Hospital Clinics:**
On the Differential Diagnosis of Swellings in the Neck (*continued*) 203
The Treatment of Migraine - - - - - - - - - 204
**A Medical Duel** - - - - - - - - - - - 202
**Sweet Charity's Guide for Christmas Givers**—
General Hospitals - - - - - - - - - - 207
Special Hospitals: Consumption—Lying-in—Epilepsy and Paralysis—Children—Women—Miscellaneous, &c. - - - 208
**Annotations.**—Diphtheria: "The Brute Force of Figures"—The Doctors and the Midwives—Anti-Toxin in the London Hospital - 205
**Notes and News** - - - - - - - - - - 620

### EXTRA SUPPLEMENT.—THE NURSING MIRROR.

**News from the Nursing World.**—Our Christmas Greeting—Our Princess—Metropolitan and Provincial Bazaars—The London Hospital—A Good Example—Questions of Interest—In South London—Nursing in Manchester—District Nursing for Daventry—Queen's Nurses in Dublin—Improved and Enlarged—Young Girls—Discounted Charity—A Notable Home—Wolverhampton—Our Christmas Competition—Short Items - - - - - - - - - - lxxvii

**Christmas Fare Two Centuries Ago** - - - - lxxix

**District Nursing in Holland** - - - - - lxxix

**How, When, and Where Nurses Spend Christmas** - lxxxi

**"Our Princess."**—H.R.H. The Princess of Wales, President of the Royal National Pension Fund - - - - lxxx

**Help the Nurses to Help the Sick** - - - - - lxxxiii

**Christmas on an Australian Fruit Farm** - - - lxxxiv

**Notes and Queries** - - - - - - - - lxxxiv

**Christmas Books** - - - - - - - - - lxxxv

**Minor Appointments** - - - - - - - - lxxxviii

**Christmas in an Irish Hospital** - - - - - - lxxxviii

**Christmas and New Year's Gifts** - - - - - lxxxix

**Appointments** - - - - - - - - - - xc

**Where to Go** - - - - - - - - - - - xc

